# Dogs (*Canis familiaris*) recognize their own body as a physical obstacle

**DOI:** 10.1038/s41598-021-82309-x

**Published:** 2021-02-18

**Authors:** Rita Lenkei, Tamás Faragó, Borbála Zsilák, Péter Pongrácz

**Affiliations:** Department of Ethology, Institute of Biology, ELTE Eötvös Loránd University, Pázmány Péter sétány 1/c, 1117 Budapest, Hungary

**Keywords:** Ecology, Evolution, Psychology, Zoology

## Abstract

Mental representations of one’s own body provide useful reference when negotiating physical environmental challenges. Body-awareness is a neuro-ontogenetic precursor for higher order self-representation, but there is a lack of an ecologically valid experimental approach to it among nonhuman species. We tested dogs (*N* = 32) in the ‘body as an obstacle’ task. They had to pick up and give an object to their owner, whilst standing on a small mat. In the test condition we attached the object to the mat, thus the dogs had to leave the mat because otherwise they could not lift the object. Dogs came off the mat more frequently and sooner in the test condition, than in the main control condition, where the object was attached to the ground. This is the first convincing evidence of body awareness through the understanding of the consequence of own actions in a species where previously no higher-order self-representation capacity was found. We urge for an ecologically valid approach, and following of bottom-up methods, in studying modularly constructed self-representation.

## Introduction

In humans, one of the first and most basic manifestations of self-representation emerges at a very early age with the understanding of the contingency between the visual and proprioceptive feedback about the position of the body. For instance, 5-month-old babies are able to distinguish their moving legs from a non-contingent video recording^1^, while later, during adulthood these cross-modal signals are still important components of the recognition of one’s own body^2^. Recent empirical results on the role of the sensory and the motor systems in self-representation became even more relevant with the new approach of embodied cognition, which emphasizes that even human cognition should be studied based on an evolutionary perspective, considering its close relationship to the physical body that interacts with the environment^3,4^.

Though currently it is widely accepted that most species possess at least some basic form of self-perception^5^, the research of self-representation in nonhuman animals still mainly concentrates on seeking the existence of such higher order cognitive capacities that normally appear towards the end of the cognitive ontogeny of self-awareness in human infants^6^. Since the famous experiment of Gallup^7^, the subsequently often criticized^8–10^ mirror mark paradigm played a pivotal role in the investigation of the animal self [e.g.^11–13^]. This approach typically lacks the possibility for formulating well-grounded ecological theories that may have driven the evolution of such capacities in nonhuman species^14^. The modular concept of self-representation^15^ allows scientists to track the evolution of this cognitive capacity, with a parallel reasoning based on forces of selection according to the ecological needs of a species.

In this study we investigated the body-awareness in a species (the dog) that has not yet been found capable of the hallmark criterion of higher-order self-representation^16^, although for both ecological and cognitive reasons, it is a likely candidate for possessing the capacity of body-awareness. Among the simplest, and very common challenges, where some sort of self-representation would be greatly adaptive for multicellular organisms with the capacity of active locomotion and a considerably complex nervous system, we should consider the encounters with either the social or the physical agents of nature. Although it is seldom assessed in nonhuman species [but see^17–19^], body awareness would be a very adaptive means to negotiate such situations when the size of an organism, or its own body, would represent an obstacle during an action^20^. Body awareness, which is “the ability to hold information about one’s own body in mind, as an explicit object, in relation to other objects in the world”^21^, can be considered as one of the fundamental building blocks of self-representation^15,22^.

The ‘own body as an obstacle’ paradigm has its connection to self-representation from the presumed knowledge about someone’s own body as an existing entity. This knowledge is originating from the visual, mechano- and proprioceptive sensory input during an action, when the task cannot be executed unless the individual moves itself away from an otherwise blocked manipulandum. Originally, based on the observation of Piaget, human toddlers were requested to hand over a blanket they were sitting on. A subject was considered as being able to understand the connection between the initial unsuccessfulness and the body as an obstacle (henceforth being ‘body-aware’), if he/she got off from the blanket. Infants younger than 18–24 months did not move off from the blanket and eventually gave up trying to finish the task.. By our knowledge, this paradigm was converted to test non-human animals only once (Asian elephants—^20^). This choice of species was motivated by the fact that elephants were earlier found to be able to ‘pass’ the mirror-mark test too^12^ (but see^23^).

Dogs have an extensive, well-proven account of complex cognitive capabilities^24^; such as empathy^25^; social learning^26^^,^^27^; theory of mind^28^; following and providing referential signals^29,30^). Based on the general complexity of dog cognition, and on the ecological conditions of the species (i.e. a highly social, mammalian predator, perfectly adapted to the most complex social environment—the anthropogenic niche), we can expect that dogs would show various components of self-representation. There are already a few indications of these (episodic-like memory—^31^, body size—^15^, recognition of own odor—^32^), and now, following the idea of a bottom-up approach, we aim at testing body awareness in dogs.

By adapting the method used by Dale and Plotnik^20^, in the main test condition (‘Test’), the object that the dogs had to pick up was attached to a mat on which the dogs were standing . We predicted that dogs would respond by leaving the mat if they understood that they could not lift the target attached to the mat because their own body impeded this. Additionally, we controlled for other mechanisms resulting in potentially similar behavioral responses. First, we tested whether the subjects are indeed able to pass an unfamiliar object to the owner (‘Unattached’). In the main control condition (‘Attached to the ground’) the dog was again standing on the mat, however, the object was fixed to the ground. This way, when the dogs tried to pick the target up, this was again impossible, however, dogs did not feel a parallel lifting force under their feet. If dogs indeed understand the connection between the obstructed removal of the object and their body as an obstacle in the Test condition, they would leave the mat sooner and more frequently than in the case of the control condition. According to this prediction, in the Attached condition, dogs would not be prompted to leave the mat while they try to lift the object that is attached to the ground. Eventually the dogs stop trying to lift the object and leave the mat. According to our prediction, in this case they would do it later and only after releasing the object. Finally, with the help of the ‘Foot discomfort’ condition (where the experimenter gently tugged the mat under the dogs’ feet by a connected rope), we planned to exclude the possibility that the subjects left the mat sooner/more often in the Test condition because they were frightened by the combined visual/physical effect they created, or just because of the sensation of the particular mat surface under their feet.

## Results

In the ‘Unattached’ and in the ‘Foot Discomfort’ conditions only a few subjects left the mat (5 from 49 dogs in both cases). In the case of the ’Foot Discomfort’ condition, we tested each of the three mat-types, and we found no difference in the frequency of the dogs leaving them (2, 2 and 1 dogs in the case of the mat from the ’Unattached’, ’Attached’, ‘Test’ conditions, respectively). With this, we could rule out that dogs would avoid particular mat surfaces more than others. To be comparable with the ‘Unattached’ and ‘Foot Discomfort’ conditions, we report only the first trial of the ‘Attached’ and ‘Test’ conditions, where 14 out of 32, and 27 out of 32, dogs left the mat, respectively.

**Figure 1:**
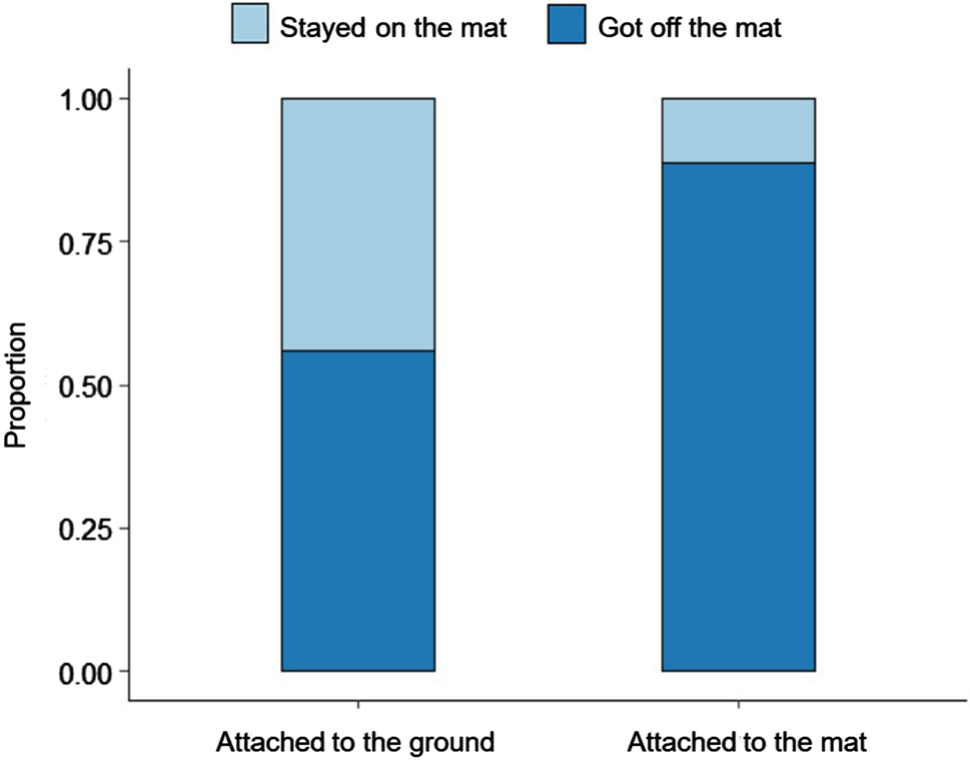
The proportion of subjects getting off or staying on the mat in the Control (‘Attached to the ground’) and ‘Test’ (‘Attached to the mat’) conditions.

By comparing the ‘Attached’ and the ‘Test’ conditions in detail, we found that each subject in all (N = 253) trials at least once tried to hold the object after the command of the owner. When comparing the frequencies of leaving the mat, we found significant effects of condition and trial (*χ*^2^(3) = 10.133; *p* = 0.017; *AIC* = 229.35). Dogs came off the mat significantly more often in the ‘Test’ condition (*β* ± SE = 2.89 ± 0.50, *z* = 5.818; *p* < 0.001, see Fig. 1) and in the case of trial 3 (*β* ± SE = 1.73 ± 0.58, *z* = 2.993; *p* = 0.003) and trial 4 as a tendency (*β* ± SE = 0.94 ± 0.54, *z* = 1.74; *p* = 0.08), than they did in trial 1. Based on the post-hoc test, the first and third trials differed from each other. We also found a significant condition effect in the case of holding the toy while the dogs left the mat (*χ*^2^(1) = 102.9; *p* < 0.001; *AIC* = 234.8). Dogs left the mat more often while still holding the toy in the ‘Test’ condition (*β* ± SE = 3.88 ± 0.52, *z* = 7.45; *p* < 0.001). Furthermore, in the case of the subjects who stepped off from the mat after leaving it, they tried or successfully passed the object to their owners in a significant proportion of the trials (77 out of 113 trials; Binomial test, p < 0.001).

In the case of the latencies, significant condition and trial effects emerged (*χ*^2^(5) = 134.84; *p* < 0.001). Dogs came off the mat sooner in the ‘Test’ condition than in the ‘Attached’ condition (exp*(β) [95%CI]* = 5.42 [4.43; 6.41]; *z* = 9.25; *p* < 0.001), and significant effects emerged in trial 2 (exp*(β) [95%CI]* = 2.19 [1.70; 2.68]; *z* = 3.49; *p* < 0.001), trial 3 (exp*(β) [95%CI]* = 2.87 [2.23; 3.51]; *z* = 4.75; *p* < 0.001) and also in the case of trial 4 (exp*(β) [95%CI]* = 3.12 [2.40; 3.85]; *z* = 4.89; *p* < 0.001). Based on pairwise comparisons, dogs left the mat with longer latencies in the first trial than in the subsequent ones. We also analyzed the latency of leaving the mat only with the hind feet (*χ*^2^(5) = 108.03; *p* < 0.001). This analysis helped rule out the potential effect of the shorter range of pulling movement in the case of ‘Attached’, as the dogs could still move off from the mat with their hind feet, while trying to solve the problem. Dogs came off the mat with their hind feet significantly sooner and more likely in the ‘Test’ condition (exp*(β) [95%CI]* = 1.85 [1.58; 2.12], *z* = 4.25; *p* < 0.001), and we also found trial effects in the second (exp*(β) [95%CI]* = 2.09 [1.68; 2.50], *z* = 3.77; *p* < 0.001), third (exp*(β) [95%CI]* = 2.60 [2.08; 3.12], *z* = 4.79; *p* < 0.001) and also in the last trial (exp*(β) [95%CI]* = 2.67 [2.13; 3.22], *z* = 4.82; *p* < 0.001). Based on the post-hoc tests, dogs left the mat later with their hind feet in the first trial than in the subsequent ones.

**Figure 2:**
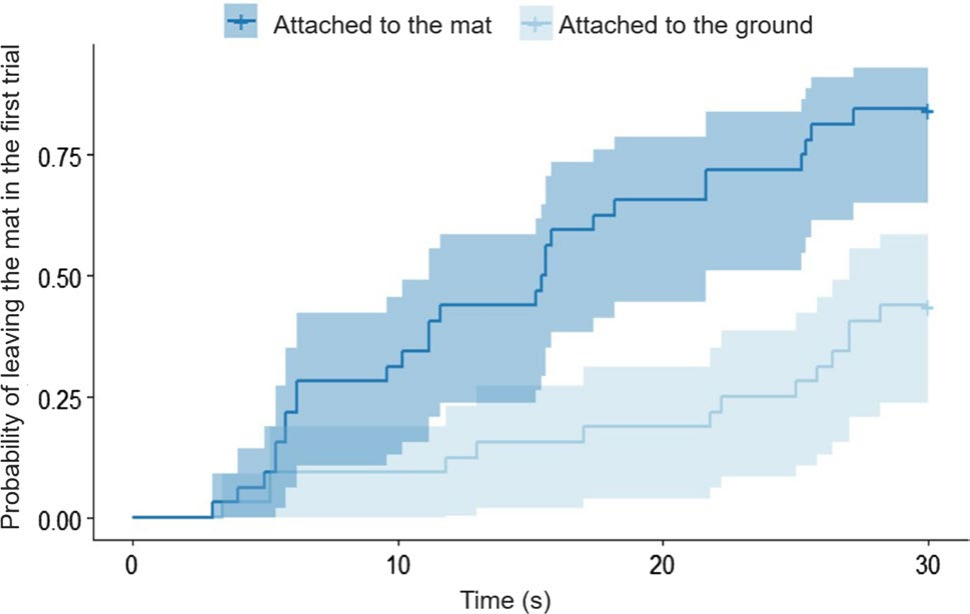
The cumulative occurrences of leaving the mat in the first trials of 'Attached' and ‘Attached to the mat’ conditions.

We also compared the dogs’ behavior between the first trials of the two conditions. We found a significant effect of testing condition (*β* ± SE = 1.99 ± 0.70, *χ*^2^(1) = 12.014; *p* < 0.001; *AIC* = 77.58) as dogs more often left the mat in the ‘Test’ condition than in the ‘Attached’ condition (*z* = 2.835; *p* = 0.005). Subjects also left the mat more often with the toy in their mouth in the ‘Test’ condition (*β* ± SE = 2.11 ± 0.72, *χ*^2^(1) = 14.684; *p* < 0.001; *AIC* = 79.977; *z* = 2.939; *p* = 0.003). Moreover, dogs left the mat sooner in the first trial of ‘Test’ condition, than in the ‘Attached’ condition (*χ*^2^(1) = 15.903; *p* < 0.001; exp*(β) [95%CI]* = 4.12 [2.67; 5.56], *z* = 4.03*; p* < 0.001, see Fig. 2). When we compared only the first trials of the ‘Test’ and ‘Attached’ conditions, we did not find significant difference between the latencies of getting off the mat with hind feet. On the other hand, this was the only comparison in the whole analysis where we found a significant order effect: (*χ*^2^(1) = 6.526; *p* = 0.01). Dogs got off from the mat with their hind feet sooner in the first trial of their second condition, regardless of whether it was an ‘Attached’ or a ‘Test’ condition (exp*(β) [95%CI]* = 2.300 [1.57; 3.03], *z* = 2.63; *p* = 0.009).

## Discussion

We found that when dogs could not pass an object to their owner, because the object was attached to the mat the dogs were standing on, they were more likely to leave the mat and leave it sooner, than in the control condition. We argue that dogs’ response in the main test can be explained based on their body awareness and the understanding of the consequences of their own actions.

The crucially important (‘Attached’) condition was missing from the work of Dale and Plotnik^20^. By including this condition, we could differentiate between two competing explanations behind the response of our subjects, which were ‘There is an obstacle’ versus ‘(My) body is an obstacle’. Dale and Plotnik (understandably) could not find a heavy enough (unmovable) object because of the strength of the elephants, and even in our study we excluded some dogs from the analysis because they pulled out the securely fixed object from the ground.

We found that all the subjects in every trial tried to lift the object at least once in both conditions, thus they had the chance to experience and understand the nature of the particular task meanwhile they tried to perform the action itself and behave accordingly. By comparing the Test and ‘Attached’ conditions, we found that dogs walked off the mat significantly more frequently, and sooner, when it was truly necessary (i.e. when their body was the obstacle). Moreover, in the Test condition, dogs more frequently walked off with the object still in their mouth than in the ‘Attached’ condition. This suggests that when a dog left the mat in the ‘Attached’ condition, it had already given up, and its departure was not an inherent component of the problem-solving sequence. While in the case of the Test condition after leaving the mat, the subjects indeed tried to pass the whole object (i.e.: ball with the mat attached) to the hand of the owner.

Still, one could argue that as the object in the ‘Attached’ condition had a limited range of movement, this may have caused dogs to leave the mat less frequently in this particular case. To exclude this explanation, we analyzed separately, the latency of stepping off with the hind feet, as this movement was not affected by the method of securing the object. As we found that the dogs left the mat sooner, and more likely with their hind feet in the Test than in the ‘Attached’ condition, this provided ample proof that they indeed started to move off earlier from the mat when it was truly needed for solving the problem (i.e. when their body was the hindering factor).

Analyzing only the first trials of the two conditions, we could determine whether the results emerged due to learning about the task or not. In the first trial of the Test condition, dogs still left the mat significantly faster and more frequently with the object in their mouth, than in the ‘Attached’ condition. Our results are in line with Dale and Plotnik^20^ as elephants also successfully completed the task above chance in their first trial.

The ‘Foot discomfort’ condition has a crucial importance in ruling out particular alternative hypotheses. In the case of the ‘body as an obstacle’ task, the participants receive feedback about the interaction between their body and the environment through visual and more importantly also through non-visual channels (i.e. proprio-/mechanoreceptors). Meanwhile dogs sensed the upwards pulling via their paws, their action also created a visual stimulation due to the bending up of the edge of the mat. One could argue that dogs simply reacted to these external stimuli during the test condition when they left the mat. However, in case of the foot discomfort condition they could sense almost exactly the same experience when the experimenter tugged the mat. Therefore, this condition showed high level similarity to the main test condition, with of course one important difference between the causation of the tugging, extrinsic when the experimenter pulled it and, intrinsic when the dog did it itself. Though the subjects sensed almost the same in both conditions, they only left the mat in the test condition when they caused the movement, and reacted differently when these stimuli were caused by their own action. Consequently, dogs might be able to represent their own actions and their consequences in their mental model and separate it from other external stimuli.

In human toddlers the onset of succeeding in the ‘body as an obstacle’ task is in parallel with the success in the well-known mirror mark test. Moore and colleagues^33^ argue that both tests are based on the knowledge of processing and representing the contingent motion relation between the self and the environment. Our results with dogs are the first indications that recognizing the body as an obstacle may exist independently from being capable of visual self-recognition. Dogs’ failure in the mirror mark test^16,34^ is likely to be explained by the inappropriate stimuli—their vision—as for them olfactory (or acoustic) stimuli might be ecologically more relevant^32,35^. In order to clarify the relationship between mirror self-recognition and the body as an obstacle, certainly further research is needed, with common ground between the two being that for passing the tasks, the subjects need to be able to hold some kind of information about themselves in their mental model. In the case of the mirror test this is information about their visual appearance, while in the body as an obstacle test, it is about one’s own actions and other physical properties of their body, as an extension. Which means that although dogs did not pass the mirror mark test, we now have evidence that they can pass the body as an obstacle test. Our results support self-representation as an array of more or less connected cognitive skills, and the presence or lack of a particular building block may vary according to the species^14,36^. All in all, this highlights the problems with the top-down approach and also emphasizes the importance for research of self-representation in other non-human species. Evolutionary based frameworks such as embodied cognition or the modular concept of self-representation^15^, would allow scientists to track the mechanism and evolution of this cognitive capacity, with a parallel reasoning, based on forces of selection according to the ecological needs of a species. Based on these concepts, two main points emerge. First, we need to establish the ecological/evolutionary background of a given species before applying empirical experiments about its cognition; and second, experiments need to be as varied as possible to determine the different aspects of a species’ self-representation.

## Methods

### Ethics

The experimental methods used in this research are in accordance with the relevant ethical regulations of the University and Hungary. Subjects of the experiments were privately owned companion dogs. All procedures involving dogs and dog owners were approved by the Ethical Committee of Eötvös Loránd University (Permission # PE/EA/2021-5/2017), and conducted in accordance with the recommendations of the Hungarian State Health and Medical Service. The owners undertook the test on a voluntary basis and they were informed that they would participate in a scientific study. All owners provided a written informed consent. Dog owners were present at the tests and they were told that they can terminate the experiment at any time when they think their dog is under unwanted stress.

### Subjects

We tested *N* = 54 adult (more than 1-year-old, mean age: 5.6 years, range: 1–11 years, see Supplementary Table 1) family dogs of various breeds and sex. We required that the subjects should be able to pass an unfamiliar object from the ground to the hand of the owner on command. Some of the subjects were recruited through social media and we also tested dogs at events, such as obedience training competitions. The reason for selecting subjects with a suitable level of a priori training was that we wanted to avoid the need for training the dogs in a specific context to pick up and hand over objects that could eventually interfere with our testing procedures. The Animal Welfare Committee of the Eötvös Loránd University accepted the experimental method (Ref. no.: PE/EA/2021-5/2017). Before the tests, dog owners were informed about the procedure, however we told them the aim of the study only after the test to avoid unwanted cueing of the subjects.

### Procedure

#### Testing site/location

**Figure 3:**
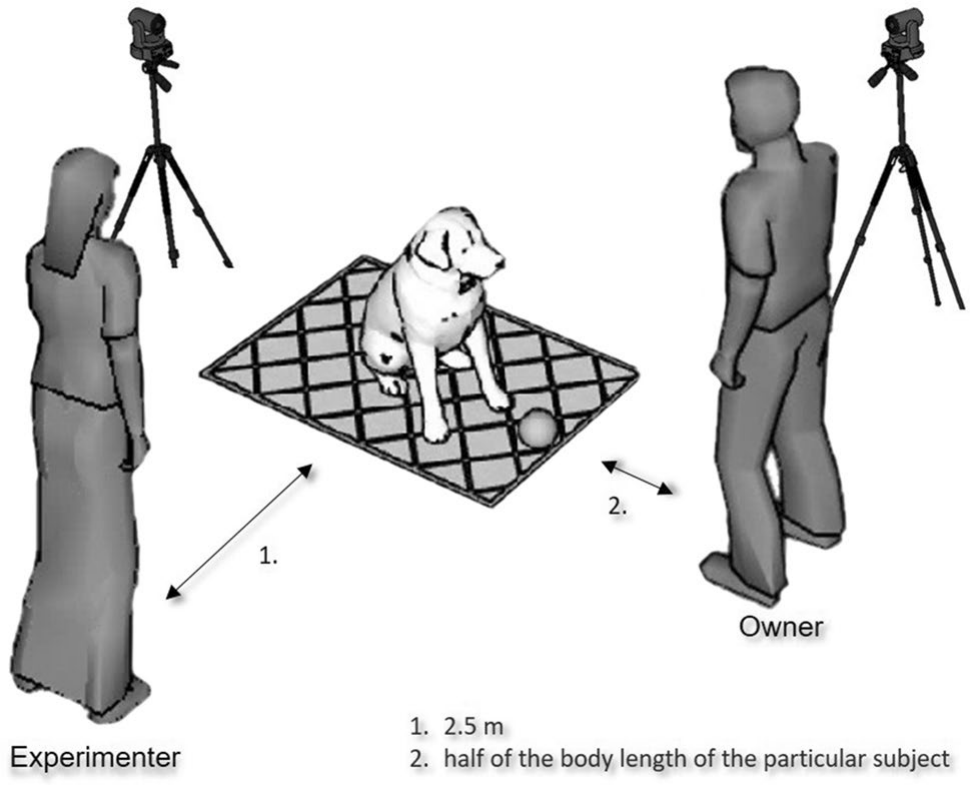
The schematic outlay of the experimental setup. The dog was asked to pick up the object (dog toy) and pass it to the hands of the owner, who stood in front of the dog, along the short side of the mat.

The tests took place at an outside grassy area. We recorded the tests with two video cameras (Sony, FDR-AX33) both standing on tripods. One camera stood 3 m behind the mat while the other was placed diagonally 3 m from its corner (Fig. 3).

#### Overview

Each subject participated in each testing condition (except those cases when we had to exclude dogs before the complete set of tests were finished—see Supplementary material for details). Each test started with the Unattached condition and then continued with the Foot Discomfort condition. If a dog did not pass either of these, we excluded him/her from the experiment. Our test was designed to be as short as possible to avoid exhaustion or loss of interest by the subjects. Therefore, we repeated the first two conditions only once (or maximum 3 times if it was needed) because we expected that dogs will rarely leave the mat. The Test and Attached conditions consisted of four repetitions each, which we performed in a randomized block order across the subjects. To avoid rapid learning, we used different unfamiliar objects and mats across conditions. In the Unattached, Attached and Test conditions the mats were made from materials of different textures and colors, but they were exactly the same size (70 × 95 cm). At the beginning of each trial the subject was sitting at the center of the rectangular mat, facing towards one of the shorter sides. Facing towards the dog, the owner (or the experimenter in the case of the Foot Discomfort condition) stood at a short step distance in front of the mat. The exact distance was always based on the size of the dog, approximately equal to half of the body length of the particular subject. Except in the case of the Foot Discomfort condition, after the signal from the Experimenter, the owner commanded the dog to pass the object to him/her which—depending on the particular condition—was attached to the mat/ground or it was unattached (thus freely movable). The owner had to command the dog in the usual way that it was trained to pass an object. We also allowed praising the dog verbally or giving treats between the trials with one stipulation: owners had to do everything the same exact way in each trial. During the trials the owner remained in the same place, although in the case of smaller dogs, he/she was allowed to bend down to receive the object from the dog. During the trials, the Experimenter stood 2.5 m from the mat at its longer side and gave the instructions the owner.

#### Detailed description of the conditions

##### Unattached object condition

This condition also served for testing whether the subject was able to pass an unfamiliar object to the owner (i.e. fulfilling the main criterion for participation). In the case of initial failure, the dog was allowed to repeat the test a maximum of two more times before being excluded from the experiment. The owner was asked to repeat the command if the dog dropped the object before successfully passing it. In this condition, the object was a soft plastic pillow.

##### Foot discomfort condition

This condition served to exclude those subjects that showed fearful behavior upon the movement of the mat, thereby avoiding causation of too much stress in particularly sensitive subjects. With the help of this condition, we could also exclude two alternative hypotheses, mainly that in the Test condition, dogs would only step off the mat to regain their balance or from discomfort or fearfulness of the moving surface, but also that the different materials of the mats had an effect on the response of the dogs. Thus, in this condition we randomly used the mats from Unattached, Attached or Test conditions. Here the dog was not requested to pass any object, we only investigated the reaction of the subjects to the sudden movements of the mat. In the beginning, the dog was commanded by the owner to sit on the mat. With the help of a rope attached to the middle of the short side of the mat, the Experimenter gently, but firmly (the front paws of the dog were lifted up a little) tugged the mat six times under the paws of the dog. We considered a dog as ‘sensitive’ if it came off the mat with three or four feet. One could argue that the exclusion of the latter subjects from further testing may impair the generalizability of our results, however, this actually strengthened our findings as we lessened the chance that some dogs would leave the mat in the Test condition just because of the startle effect caused by its movement.

##### Attached to the ground condition

In this control condition, we investigated whether dogs were able to differentiate between cases when their body obstructed the lifting of the object or when lifting was impeded for other reasons. Thus, in this condition the object (a ball made of knotted rope) was attached to the ground with the help of a hook along the end of the short side of the mat. As the ball was tied to the hook, it had an approximately 5 cm range to move as the dog pulled it and the knot became tighter. The trial started when the Experimenter gave the signal to begin, the owner then commanded the dog to give the ball to him/her. The trial was over when the dog stepped off the mat with all four feet or after a maximum of 30 s. It is important to mention that visually it was impossible to tell initially, whether the object was attached to the mat only or to the ground across the edge of the mat. If dogs would be able to distinguish between the unsolvable (Attached to the ground) and the Test condition by simply looking at the equipment, one could argue that subjects may react differently to the two conditions because they decided a priori that the task was impossible to accomplish in the ‘Attached to the ground’ condition.

##### Test condition: attached to the mat

The procedure was identical to the Attached control condition, except that the object (a knotted rope toy) was attached to the middle of the short edge of the mat.

### Data collection

The behavior of the subjects was coded from the video segments with the help of Solomon Coder (beta 17.03.22 copyright by András Péter). We measured the latency of leaving the mat and coded whether dogs left the mat (yes/no). Both variables involved a complete leaving of the mat, meaning that the dog stepped off with all four feet. We also coded whether the object was in the mouth of the dog while it was stepping off the mat (yes/no) and whether the dog tried to pass the object to the owner after stepping off the mat (yes/no) in the Test condition within 10 s. For more detailed information about the dynamics of leaving the mat, we also coded the latency of stepping off the mat with the hind feet only. To check the coding procedure’s reliability, an independent observer coded *N* = 10 video segments. Based on Spearman’s Rho coefficient, the coding method proved to be reliable in the case of each variable (latency of leaving the mat with the hind feet: *rs* = 0.998; *p* < 0.001; latency of leaving the mat with the front feet: *rs* = 0.994; *p* < 0.001; holding the toy meanwhile leaving the mat: *rs* = 0.843; *p* < 0.001).

### Statistical analysis

The statistics were made in R environment (R Core Team, 2016) with coxme and lme4 packages. The binary variables were analyzed with generalized linear mixed model (GzLMM) using binomial distribution (glm function) and forward model selection. We ran step-wise forward model selection based on AIC values. The model with the lowest AIC value was kept and we considered a model better if the δ AIC value was equal or greater than 2. In case of pairwise comparisons, we ran Tukey post hoc tests (emmeans). For the latencies, we used Mixed Effects Cox Regression (coxme function). We report the results of the final models.

### Exclusions

Five out of 54 subjects did not give the unfamiliar object to the owner’s hand in the Unattached condition. From the remaining 49 subjects five dogs were excluded after the ‘Foot Discomfort’ condition because they were sensitive to the moving mat. An additional six subjects were excluded from the remaining 44 dogs in the ‘Attached’ condition because they were eventually able to remove the anchored object from the ground. Finally, 6 out of 38 subjects were excluded because of technical reasons (e.g.: the dog was inattentive; the owner did not follow the instructions, problems with the video recording). All in all, *N* = 32 subjects took part in all of the conditions.

## Supplementary Information

Supplementary Information 1.

Supplementary Video S1.

## Data Availability

Data will be available upon request.

## References

[1] Bahrick LE, Watson JS (1985). Detection of intermodal proprioceptive–visual contingency as a potential basis of self-perception in infancy. Dev. Psychol..

[2] Van Den Bos E, Jeannerod M (2002). Sense of body and sense of action both contribute to self-recognition. Cognition.

[3] Wilson M (2002). Six views of embodied cognition. Psychon. B. Rev..

[4] Smith L, Gasser M (2005). The development of embodied cognition: Six lessons from babies. Artif. life.

[5] Shettleworth, S. J. *Cognition, Evolution, and Behavior*. Oxford University Press.

[6] Kohda M, Hotta T, Takeyama T, Awata S, Tanaka H, Asai JY, Jordan AL (2019). If a fish can pass the mark test, what are the implications for consciousness and self-awareness testing in animals?. PLoS Biol.

[7] Gallup GG (1970). Chimpanzees: Self-recognition. Science.

[8] Epstein R, Lanza RP, Skinner BF (1981). "Self-awareness" in the pigeon. Science.

[9] Heyes CM (1995). Self-recognition in primates: Further reflections create a hall of mirrors. Anim. Behav..

[10] Suddendorf T, Butler DL (2014). Response to Gallup et al.: Are rich interpretations of visual self-recognition a bit too rich?. Trends. Cogn. Sci..

[11] Reiss D, Marino L (2001). Mirror self-recognition in the bottlenose dolphin: A case of cognitive convergence. Proc. Natl. Acad. Sci. USA.

[12] Plotnik JM, De Waal FB, Reiss D (2006). Self-recognition in an Asian elephant. Proc. Natl. Acad. Sci. USA.

[13] Prior H, Schwarz A, Güntürkün O (2008). Mirror-induced behavior in the magpie (*Pica pica*): Evidence of self-recognition. PLoS Biol.

[14] Bekoff M, Sherman PW (2004). Reflections on animal selves. Trends Ecol. Evol..

[15] Lenkei R, Faragó T, Kovács D, Zsilák B, Pongrácz P (2019). That dog won’t fit: Body size awareness in dogs. Anim. Cogn.

[16] Zazzo R (1979). Des enfants, des singes et des chiens devant le miroir. Rev. Psychol. Appl..

[17] Cuthill I, Guilford T (1990). Perceived risk and obstacle avoidance in flying birds. Anim. Behav..

[18] Khvatov IA, Sokolov AY, Kharitonov AN (2019). Snakes *Elaphe radiata* may acquire awareness of their body limits when trying to hide in a shelter. Behav. Sci..

[19] Maeda, T. & Fujita, K. Do dogs (*Canis familiaris*) recognize their own body size? In *Proceedings of the 2nd Canine Science Forum, Vienna, Austria*, 52 (2010).

[20] Dale R, Plotnik JM (2017). Elephants know when their bodies are obstacles to success in a novel transfer task. Sci. Rep..

[21] Brownell CA, Zerwas S, Ramani GB (2007). “So big”: The development of body self-awareness in toddlers. Child Dev..

[22] Povinelli DJ, Cant JG (1995). Arboreal clambering and the evolution of self-conception. Q. Rev. Biol..

[23] Povinelli DJ (1989). Failure to find self-recognition in Asian elephants (*Elephas maximus*) in contrast to their use of mirror cues to discover hidden food. J. Comp. Psychol..

[24] Topál J, Miklósi Á, Gácsi M, Dóka A, Pongrácz P, Kubinyi E, Virányi Z, Csányi V (2009). The dog as a model for understanding human social behaviour. Adv. Stud. Behav..

[25] Sanford EM, Burt ER, Meyers-Manor JE (2018). Timmy’s in the well: Empathy and prosocial helping in dogs. Learn. Behav..

[26] Pongrácz P, Bánhegyi P, Miklósi Á (2012). When rank counts—dominant dogs learn better from a human demonstrator in a two-action test. Behaviour.

[27] Huber L, Popovová N, Riener S, Salobir K, Cimarelli G (2018). Would dogs copy irrelevant actions from their human caregiver?. Learn. Behav..

[28] Virányi ZS, Topál J, Miklósi Á, Csányi V (2006). A nonverbal test of knowledge attribution: A comparative study on dogs and children. Anim. Cogn..

[29] Polgárdi R, Topál J, Csányi V (2000). Intentional behaviour in dog-human communication: An experimental analysis of “showing” behaviour in the dog. Anim. Cogn..

[30] Pongrácz P, Hegedüs D, Sanjurjo B, Kővári A, Miklósi Á (2013). “We will work for you”—Social influence may suppress individual food preferences in a communicative situation in dogs. Learn. Motiv..

[31] Fugazza C, Pogány Á, Miklósi Á (2016). Recall of others’ actions after incidental encoding reveals episodic-like memory in dogs. Curr. Biol..

[32] Horowitz A (2017). Smelling themselves: Dogs investigate their own odours longer when modified in an “olfactory mirror” test. Behav. Proc..

[33] Moore C, Mealiea J, Garon N, Povinelli DJ (2007). The development of body self-awareness. Infancy.

[34] Howell TJ, Bennett PC (2011). Can dogs (*Canis familiaris*) use a mirror to solve a problem?. J. Vet. Behav..

[35] Bekoff M (2002). Awareness: Animal reflections. Nature.

[36] Kaplan JT, Aziz-Zadeh L, Uddin LQ, Iacoboni M (2008). The self across the senses: An fMRI study of self-face and self-voice recognition. Soc. Cogn. Affect. Neur..

