# Supplementary material for: Dogs (Canis familiaris) recognize their own body as a physical obstacle
Source: Sci Rep. 2021 Feb 18;11:2761. doi: 10.1038/s41598-021-82309-x (PMC7893002; doi:10.1038/s41598-021-82309-x)
Supplement: Supplementary file 1 — Supplementary Information 1. [file 41598_2021_82309_MOESM1_ESM.docx]

**dogs (*Canis familiaris*) recognize their own body as a physical obstacle**

Rita Lenkei, Tamás Faragó, Borbála Zsilák & Péter Pongrácz

Supplementary Material

| **Dog ID** | **Breed** | **Sex** | **Age (years)** | **Rep. status** | **Mat type in the Foot Condition** | **First condition** | **Status** | **Reason of exclusion** |
| --- | --- | --- | --- | --- | --- | --- | --- | --- |
| 1 | Mixed | F | 5.0 | neutered | 1 | test |  |  |
| 2 | Rhodesian Ridgeback | M | 12.0 | neutered |  |  | EX | could not pass the object |
| 3 | Sheltie | M | 6.0 | neutered | 1 | test |  |  |
| 4 | Miniature Schnauzer | F | 8.0 | neutered | 1 | attached to ground |  |  |
| 5 | Giant Schnauzer | M | 3.0 | Intact | 2 | attached to ground | EX | pulled out |
| 6 | Labrador Retriever | F | 4.0 | neutered | 1 | test |  |  |
| 7 | Golden Retriever | F | 5.0 | Intact | 3 | test |  |  |
| 8 | Labrador | M | 2.0 | intact | 1 | test | EX | error in the method |
| 9 | Miniature Poodle | M | 4.0 | neutered | 2 | test |  |  |
| 10 | Giant Schnauzer | M | 3.0 | neutered | 2 | test |  |  |
| 11 | Mixed | M | 7.0 | neutered | 3 | test | EX | sensitive |
| 12 | German Wirehaired Pointer | M | 5.0 | neutered | 3 | test | EX | pulled out |
| 13 | Mixed | M | 10.0 | intact | 3 | attached to ground |  |  |
| 14 | Groenendael | F | 11.0 | neutered | 3 | attached to ground |  |  |
| 15 | Mixed | F | 4.0 | neutered | 3 | test |  |  |
| 16 | Labrador Retriever | F | 6.0 | neutered | 1 | test |  |  |
| 17 | Border Collie | M | 1.4 | intact | 3 | test |  |  |
| 18 | Border Collie | F | 3.0 | intact | 3 | attached to ground |  |  |
| 19 | Hungarian Vizsla | F | 8.0 | neutered | 2 | test | EX | error in the method |
| 20 | Mixed | F | 4.5 | intact | 3 |  | EX | could not pass the object |
| 21 | Labrador Retriever | M | 10.0 | intact | 3 | attached to ground |  |  |
| 22 | Malinois | M | 7.5 | intact | 2 | test | EX | pulled out |
| 23 | Australian Shepherd | M | 7.0 | neutered | 1 | attached to ground |  |  |
| 24 | Malinois | M | 8.0 | intact | 2 |  | EX | could not pass the object |
| 25 | Mudi | F | 4.5 | neutered | 1 | test |  |  |
| 26 | Malinois | F | 4.0 | intact | 2 | attached to ground | EX | pulled out |
| 27 | Malinois | F | 2.0 | intact |  |  | EX | could not pass the object |
| 28 | German Shepherd | F | 4.0 | neutered | 1 | test | EX | error in the method |
| 29 | Hungarian Vizsla | M | 8.0 | intact | 2 | test | EX | interrupted by the owner |
| 30 | Poodle | M | 5.0 | intact | 2 | attached to ground | EX | sensitive |
| 31 | Mixed | F | 11.0 | intact | 2 | attached to ground |  |  |
| 32 | Border Collie | F | 2.5 | neutered | 1 | attached to ground |  |  |
| 33 | Mixed | M | 4.0 | neutered | 2 |  | EX | sensitive |
| 34 | Mixed | M | 10.5 | neutered | 1 | attached to ground |  |  |
| 35 | Mixed | F | 5.5 | intact | 3 | attached to ground |  |  |
| 36 | Beagle | F | 10.0 | intact | 3 | test | EX | error in the method |
| 37 | Labradoodle | M | 1.0 | neutered | 2 | attached to ground |  |  |
| 38 | Mixed | F | 3.0 | neutered | 3 | attached to ground |  |  |
| 39 | Mixed | M | 8.0 | neutered | 1 | test | EX | sensitive |
| 40 | Hungarian Vizsla | F | 7.0 | neutered | 2 | attached to ground |  |  |
| 41 | Border Collie | F | 7.0 | intact |  |  | EX | could not pass the object |
| 42 | Mixed | F | 11.0 | neutered | 3 | test |  |  |
| 43 | Mixed | F | 1.5 | intact | 3 | attached to ground | EX | error in the method |
| 44 | Dachshund | M | 8.0 | intact | 2 | test |  |  |
| 45 | Poodle | M | 3.0 | neutered | 3 | attached to ground |  |  |
| 46 | Patterdale Terrier | M | 5.0 | intact | 1 | attached to ground |  |  |
| 47 | Golden Retriever | M | 7.0 | intact | 3 | test | EX | sensitive |
| 48 | Malinois | F | 2.0 | intact | 2 | attached to ground |  |  |
| 49 | Border Collie | F | 3.0 | neutered | 1 | attached to ground |  |  |
| 50 | Malinois | M | 2.5 | intact | 2 | attached to ground | EX | pulled out |
| 51 | Mixed | F | 3.5 | neutered | 1 | attached to ground |  |  |
| 52 | Mixed | M | 4.0 | intact | 2 | attached to ground |  |  |
| 53 | Mixed | M | 7.0 | intact | 2 | test | EX | pulled out |
| 54 | Border Collie | F | 3.0 | neutered | 1 | attached to ground |  |  |

Supplementary Table 1 The basic information of the subjects and their participation in the testing conditions. In the case of a subject that has been excluded from further testing, the reason is indicated in the last column. F=female, M=male, EX=excluded
